# Chromosome-scale genome assembly of the brown anole (*Anolis sagrei*), an emerging model species

**DOI:** 10.1038/s42003-022-04074-5

**Published:** 2022-10-25

**Authors:** Anthony J. Geneva, Sungdae Park, Dan G. Bock, Pietro L. H. de Mello, Fatih Sarigol, Marc Tollis, Colin M. Donihue, R. Graham Reynolds, Nathalie Feiner, Ashley M. Rasys, James D. Lauderdale, Sergio G. Minchey, Aaron J. Alcala, Carlos R. Infante, Jason J. Kolbe, Dolph Schluter, Douglas B. Menke, Jonathan B. Losos

**Affiliations:** 1 Department of Biology, Center for Computational and Integrative Biology, Rutgers University–Camden, Camden, NJ USA; 2 Department of Genetics, University of Georgia, Athens, GA USA; 3 Department of Biology, Washington University in St. Louis, St. Louis, MO USA; 4 Department of Ecology and Evolutionary Biology and Biodiversity Institute and Natural History Museum, University of Kansas, Lawrence, KS USA; 5 Max Perutz Labs, Medical University of Vienna, Vienna, Austria; 6 School of Informatics, Computing and Cyber Systems, Northern Arizona University, Flagstaff, AZ USA; 7 Institute at Brown for Environment and Society, Brown University, Providence, RI USA; 8 Department of Biology, University of North Carolina Asheville, Asheville, NC USA; 9 Department of Biology, Lund University, 223 62 Lund, Sweden; 10 Department of Cellular Biology, University of Georgia, Athens, GA USA; 11 Department of Integrative Biology, University of Colorado Denver, Denver, CO USA; 12 Department of Biological Sciences, University of Rhode Island, Kingston, RI USA; 13 Department of Zoology, University of British Columbia, Vancouver, Canada; 14 Living Earth Collaborative, Washington University in St. Louis, St. Louis, MO USA

**Keywords:** Evolution, Genomics

## Abstract

Rapid technological improvements are democratizing access to high quality, chromosome-scale genome assemblies. No longer the domain of only the most highly studied model organisms, now non-traditional and emerging model species can be genome-enabled using a combination of sequencing technologies and assembly software. Consequently, old ideas built on sparse sampling across the tree of life have recently been amended in the face of genomic data drawn from a growing number of high-quality reference genomes. Arguably the most valuable are those long-studied species for which much is already known about their biology; what many term emerging model species. Here, we report a highly complete chromosome-scale genome assembly for the brown anole, *Anolis sagrei* – a lizard species widely studied across a variety of disciplines and for which a high-quality reference genome was long overdue. This assembly exceeds the vast majority of existing reptile and snake genomes in contiguity (N50 = 253.6 Mb) and annotation completeness. Through the analysis of this genome and population resequence data, we examine the history of repetitive element accumulation, identify the X chromosome, and propose a hypothesis for the evolutionary history of fusions between autosomes and the X that led to the sex chromosomes of *A. sagrei*.

## Abstract (web summary)

A highly-complete chromosome-scale genome assembly of the brown anole, *Anolis sagrei*, provides insight into the evolution of sex chromosomes and is a crucial resource for this model lizard species.

## Introduction

Recent breakthroughs in high-throughput sequencing, coupled with the creation of long-distance scaffolding libraries, have ushered in an era of ever-improving quality and quantity of genome assemblies. Genome assemblies now routinely span entire chromosomes and include data from formerly impenetrable genomic regions^1–3^. In turn, these assemblies have enabled increasingly sophisticated genomic analyses of organismal traits and behaviors, and the evolutionary and ecological implications of the interactions of genomes and the environment. Massive reductions in the cost of genome sequencing and assembly have allowed non-model and emerging model species to become genome-enabled, a neologism indicating that genomic information has become available for the species. Observations from these new assemblies have provided fresh insights into core biological processes. For example, our understanding of recombination^4^, repetitive genetic elements^5–7^, chromosome evolution^8–10^ and dosage compensation^4,11,12^ have all been fundamentally changed due to results made possible by recent genome assemblies of non-traditional model species.

Efforts are underway to generate thousands of new genome assemblies for species across the tree of life^13–15^. However, our understanding of the biology of most species on earth remains sorely lacking – limiting the inferential power gained by the addition of genomic data. In contrast, those species for which the existing organismal literature is vast are particularly primed for the generation of high-quality genome assemblies because new discoveries concerning the genetic basis of organismal traits await only the addition of a reference genome that include nearly all genomic features present in the species (completeness) and assembled into scaffolds that approach the size of the species’ actual chromosomes.

While the production of highly contiguous genome assemblies is a technological achievement, the long-term value of these assemblies is that they serve as critical tools in the advancement of biological research. Evolutionary genomic techniques such as quantitative trait locus mapping or genome-wide association studies enable careful examination of the genetic basis of organismal traits, but these rely on the accurate positioning and orientation of genomic features to connect genotype to phenotype. Improved contiguity of genome assemblies therefore paved the way for a finer and more accurate understanding of the genomic basis of organismal traits.

Further, understanding the evolutionary history of a species’ chromosomes similarly requires highly complete genome assemblies since only with these data can chromosomal sequence homology be reliably inferred^16,17^. While cytogenetics opened the door to inferring evolutionary transitions in chromosome complement well over 100 years ago^18^, only recently through genome sequencing have the evolutionary drivers and consequences of these changes begun to be understood. While the first wave of genome assemblies lacked the contiguity and completeness to fully determine syntenic relationships between species, new chromosome-scale assemblies now enable the rigorous study of chromosome evolution.

Finally, population genomic scans also benefit from improved contiguity. For example, recent selective sweeps leave patterns of reduced genetic diversity in the genomic regions surrounding the selected variant^19,20^. Many methods to detect recent selection rely on these patterns but poorly constructed genome assemblies can separate that signal onto separate scaffolds and limit our ability to detect these patterns.

## The brown anole

**Fig. 1:**
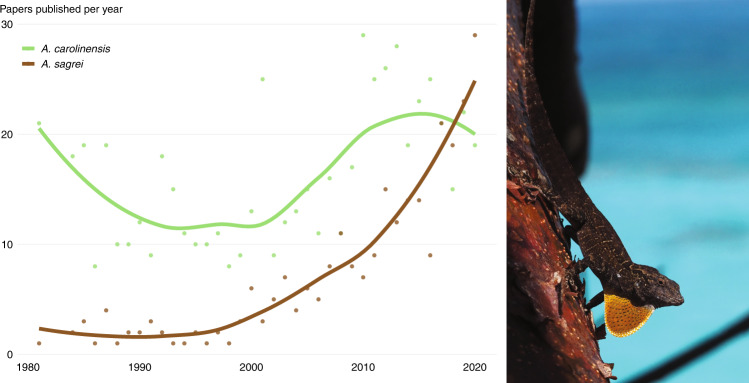
The rise of *Anolis sagrei*. Over the past two decades research interest in *Anolis sagrei* (pictured at right) has grown substantially and recently surpassed that of *A. carolinensis*, which for many decades served as the model reptile species in biological research. Queries for each specific epithet were performed in the indexed Titles and Abstracts on https://www.dimensions.ai (accessed May 2021). Underlying data and approach appear in Supplementary Data 1 and Supplementary Note 1.

*Anolis* lizards (anoles) comprise over 400 small- to medium-sized lizard species distributed throughout the continental neotropics of South, Central, and North America, and across islands in the West Indies and eastern Pacific Ocean^21^. The green anole (*Anolis carolinensis*) was the first reptile to have its full genome assembled^22^. While it was sequenced using first-generation genome sequencing technologies over 10 years ago, it remains one of the best assembled and annotated reptile genomes and by far the most complete and contiguous assembly within the genus *Anolis*. It was selected for genome sequencing due to many decades of biomedical research–especially epidemiology and neurobiology–using this species as a model. Recently, a second species, the brown anole (*Anolis sagrei*), has surpassed the green anole in publications per year (Fig. 1, Supplementary Data 1) and is considered an emerging model species for numerous fields^23–26^.

*Anolis sagrei* is a medium-sized insectivorous lizard most commonly found on the ground and perched low on the trunks of trees^27^. Although it first arose on Cuba^28^, the species now has the largest native range of any anole with natural diaspora populations found across islands of the northern Caribbean as well as coastal areas of Mesoamerica^28,29^. It is also a prolific invader with non-native populations established on many additional islands in the West Indies^30,31^, multiple locations in North^32^, South^33^ and Central America^34^, as well as remote islands of the central Atlantic Ocean^35,36^, Hawaii^37^, Taiwan^38^, Asia, Europe, and the Middle East.

A recent analysis of genome-scale sequence data revealed that *A. sagrei* evolved on Cuba toward the end of the Miocene^28^. Two major lineages are present on East and West Cuba, and although they are not geographically separate, they represent ancient evolutionary separation and probable secondary contact. Both lineages have given rise to diaspora populations that have colonized other island groups. The western Cuba lineage colonized the Bahamas Archipelago in both the Pliocene and Pleistocene, while the eastern lineage colonized the Cayman Archipelago, the Swan Islands, Mesoamerica, and Jamaica at different periods during the Pleistocene^28^. These diaspora lineages, despite different evolutionary backgrounds and divergence times, have evolved a similar suite of phenotypic traits such that Cuban *A. sagrei* can be distinguished from diaspora *A. sagrei* using both genetic and phenotypic characters^28^. This suggests that the species has responded to presumably similar evolutionary selective pressures when colonizing islands elsewhere in the Caribbean. Notably, both relatively larger body size and increased number of subdigital lamellar scales appear to be features of diasporic lineages, although it is currently unknown whether similar genomic changes are responsible for these outcomes.

Multiple factors have led to the rapidly increasing use of *A. sagrei* for research in evolution and ecology. These include its wide natural and invasive ranges, its high local abundance, and the fact that this species is amenable to captive treatments, including breeding and rearing in a laboratory setting^39,40^. As a result, the brown anole has become a broadly used system in natural environments as well as in the lab^41^ to study evolutionary ecology^42–44^, behavior^24,45^, development^26,46–49^, reproductive isolation^50^, sexual selection^25,51–54^, biological invasions^23,55–57^, and adaptation^55,58,59^. However, the lack of a reference genome has made it challenging to connect this depth of knowledge of brown anole phenotypes to their underlying genetic architecture. Despite this limitation, the brown anole has been at the forefront of new techniques, including chromosome microdissection and sequencing^60,61^ and recently became the first reptile to successfully undergo CRISPR-Cas9 genome editing^62^. This last breakthrough begs for the production of a high-quality reference genome to establish the brown anole as a fully-fledged model organism.

Here, we report a highly complete and contiguous genome assembly of a single female brown anole (*Anolis sagrei ordinatus*) from the Central Bahamas. We supplement this assembly with evidence-based and ab initio gene model annotation, repetitive element identification and analysis, and a map of segregating genetic diversity. Finally, we build on existing research to confirm the identity of the *A. sagrei* X chromosome and identify patterns of the evolution of the *A. sagrei* X chromosome relative to its counterparts in the *A. carolinensis* genome.

## Results and discussion

**Fig. 2:**
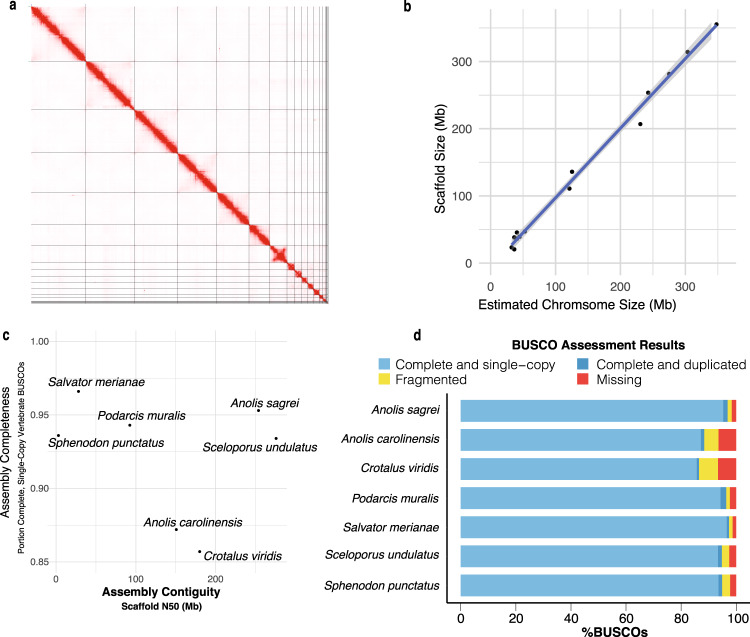
Contiguity and completeness of *Anolis sagrei* and other lepidosaur assemblies. **a** Link density histogram of the AnoSag2.1 assembly depicting the mapping location of HiC read pairs. Increasing intensity of red indicated more read pairs map to a particular coordinate. White corresponds to no reads mapping and the reddest cells have 10,000 read pairs mapping to that coordinate. **b** The scaffold sizes of the AnoSag2.1 assembly are highly correlated with chromosome sizes estimated from karyotype imaging. Raw data appear in Supplementary Data 15**c** Scatterplot of recent high-quality lepidosaur genome assemblies. **d** BUSCO assessment of assembly completeness for AnoSag2.1 and other selected lepidosaur assemblies. Underlying data appear in Supplementary Data 3.

We created a highly complete and contiguous draft genome assembly of *A. sagrei* through multiple rounds of iterative improvement. Our initial assembly using only Illumina whole-genome shotgun sequences and assembled using meraculous^63^ produced a largely fragmented assembly, which was incomplete in terms of gene content and total size (Supplementary Data 2). Subsequent scaffolding performed in HiRise^64^ using Chicago and HiC proximity ligation libraries substantially improved both contiguity and completeness, but the assembly remained substantially smaller (1.6 Gb) than the 1.8 Gb assembly of *A. carolinensis* and a genome size estimate of 1.89 Gb for *A. sagrei* based on fluorescence cytophotometry^65^. We further refined the *A. sagrei* genome assembly by improving contig size with error-corrected PacBio long reads and re-scaffolding in HiRise. The addition of these data resulted in a far more contiguous and complete assembly, the size of which (1.93 Gb) very closely matches the expected genome size for this species (Supplementary Data 2). Analysis of HiC mapped read link density using Juicer v1.6^66^ revealed that two chromosomes had been artificially joined during the assembly process. Using evidence from Illumina short-read, RNA-Seq, and PacBio data (see Methods) we corrected this misjoin resulting in the current *A. sagrei* assembly version (hereafter, AnoSag2.1). A link density histogram of HiC read pairs mapped to the AnoSag2.1 assembly does not show evidence of remaining misjoins (Fig. 2a). The mitochondrial genome was not captured in this assembly but was recovered through a combination of circularized *de novo* assembly and identification of mitochondrial sequence in an error-corrected PacBio read. The consensus of these two approaches yields a 17,535 bp assembly with the 13 genes, 22 tRNAs, and two ribosomal RNAs expected for vertebrates and with identical gene ordering to the *A. carolinensis* mitochondrial genome.

### Contiguity and Completeness

Our AnoSag2.1 assembly has a scaffold N50 of 253.6 Mb, which is 1.6 times as contiguous as *Anolis carolinensis*, the longtime standard bearer for reptile genome assemblies^22^. The four largest AnoSag2.1 scaffolds comprise more than 50% of the genome assembly. The *A. sagrei* karyotype contains 14 chromosomes: six macrochromosomes, seven microchromosomes and the intermediately sized X chromosome. Multiple lines of evidence suggest that our assembly recovers each of these chromosomes as the 14 largest scaffolds. First, the 14 largest scaffolds in AnoSag2.1 comprise 99.1% of the assembled genome sequence. Furthermore, a large drop-off in scaffold size occurs after the last putative chromosome – scaffold 14 is over 20 Mb in size where the next largest scaffold is two orders of magnitude smaller (scaffold 15; 131 kb). Finally, the AnoSag2.1 scaffold sizes are highly correlated (*r*^2^ = 0.996, *p* < 2.2×10^−16^) with chromosome sizes estimated using a published karyotype^67^ of this species (Fig. 2b).

We assessed completeness of our assembly using BUSCO 5.0.0 which tests for the presence of a curated set of 3,354 protein-coding genes known to be present in single copy across vertebrate genomes (vertebrata_odb10). Of these genes, 3,197 (95.3%) are present in full length and found to be single-copy in our assembly. The AnoSag2.1 assembly is missing only 1.6% of the genes from this set. Our assembly exceeds most other Lepidosauria (lizards, snakes, and tuatara) genome assemblies in contiguity and completeness^4,22,68–71^ (Supplementary Data 3). Only the Argentine black and white tegu^70^ (*Salvator merianae*) exceeds our assembly in BUSCO completeness but is substantially less contiguous (Fig. 2c). The eastern fence lizard^71^ (*Sceloporus undulatus*) is slightly more contiguous than our assembly but less complete. These two genomes stand apart from other recent lepidosaur genome assemblies in being both highly complete and contiguous (Fig. 2b, c, d). Variation in both summary statistics can be partially attributed to the specific biology of a sequenced species. For example, two fully assembled genomes of equal total size will have different N50 values based on the size distribution of their chromosomes alone. Similarly, lineage-specific loss or duplication of genes will influence BUSCO completeness scores even if all genes in a species are perfectly annotated. While it is not possible to determine the exact reason for variation in completeness and contiguity our assembly likely benefitted from 1) the relatively large macrochromosomes in *A. sagrei*, 2) the combined use of short and long read sequencing with Chicago and HiC long-distance interaction data and 3) reduced heterozygosity in the individual sequenced (see Methods).

### Annotation statistics

We performed an automated annotation of our assembly using Braker v2.0.5^72^ followed by manual curation of all gene models. This effort resulted in a final set of 20,033 genes comprising 902 Mb (47% of the final assembly length). Most gene models (94%) contain more than one exon and all exons summed account for a total length of 55 Mb, or about 3% of the assembly (Supplementary Data 4). Start codons are annotated for 99.7% of all gene model and the same percentage have stop codons annotated (although not all within the same genes). One measure of the completeness of a gene annotation is to contrast BUSCO analysis of the annotated exome with a BUSCO analysis of the entire genome. A highly complete exome should recover all the BUSCOs found in the full genome sequence. This analysis suggests our annotation captures most of the genes found in the homology-based BUSCO search – 96.9% of vertebrate universal single-copy orthologs were found to be complete via homology search of the entire genome sequence versus 94.5% found within the gene models in our annotation. While our annotation is quite complete, its completeness is slightly exceeded by the tegu, fence lizard, and wall lizard annotations (Supplementary Data 3). Possible explanations for this include differences in the annotation pipeline and RNA sequencing evidence used to support gene annotation.

### Repetitive element landscape

We estimated 51.7% of the *Anolis sagrei* genome to be repetitive, compared with 32.9% for *A. carolinensis*, including 39.3% comprised of known interspersed repeats, and 10% unclassified repeats. The larger proportion of repeats of unknown classification in the *A. sagrei* assembly compared to that of *A. carolinensis* may represent centromeric repeats captured by long reads. Both genomes contained a diversity of transposable elements, including short interspersed elements (SINEs), long interspersed nuclear elements (LINEs), long terminal repeat retrotransposons (LTRs), and DNA transposons (Table 1). *Anolis sagrei* contained a higher proportion of LINEs and DNA transposons, whereas *A. carolinensis* contained relatively more LTR retrotransposons.

**Table 1:** Repetitive Elements.

Repeat Class	*Anolis sagrei*		*Anolis carolinensis*	
	Length occupied (bp)	Percent of Genome	Length occupied (bp)	Percent of Genome
SINEs	55,937,322	2.90	75,887,012	4.22
LINEs	440,199,767	22.85	234,058,101	13.01
LTR elements	41,622,585	2.16	84,049,288	4.67
DNA transposons	218,634,812	11.35	157,677,814	8.76
Unclassified	191,828,398	9.96	34,170,372	1.90
Total Interspersed Repeats	948,222,884	49.22	585,842,587	32.56
Total Repeats	995,795,378	51.69	591,836,683	32.90

Comparison of the interspersed repeat contents of *Anolis sagrei* and *Anolis carolinensis.*

**Fig. 3:**
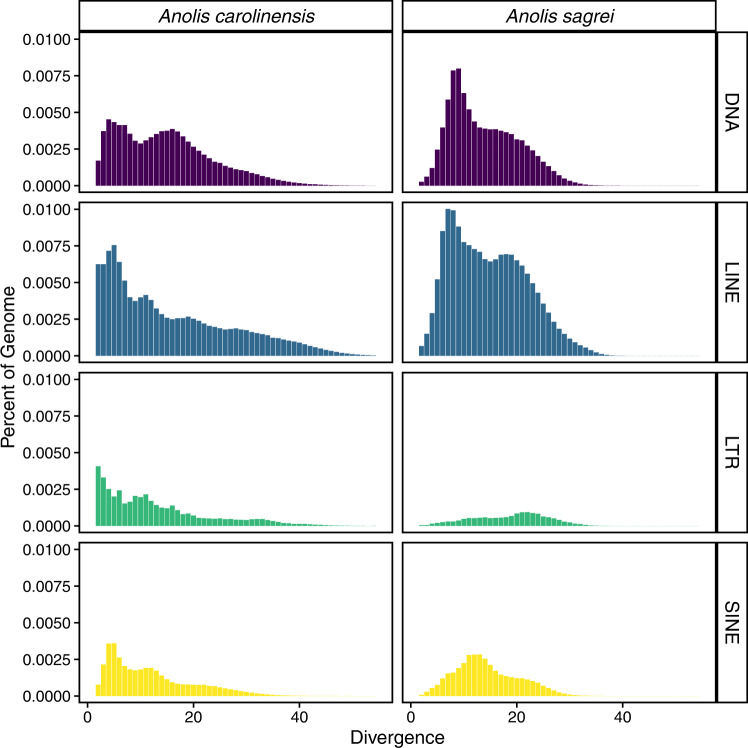
Comparison of repeat landscapes for the classes of transposable elements in *Anolis carolinensis* and *Anolis sagrei*. The proportion of the genome consisting of transposable element insertions (short interspersed elements=SINE, long interspersed elements=LINE, long terminal repeat retrotransposons=LTR, and DNA transposons=DNA) of different ages according to their Kimura 2-parameter divergence from consensus. Older insertions are more divergent. Underlying data appear in Supplementary Data 5 and 6.

We examined the age distribution of repeats in each genome, or its repeat landscape, by comparing the proportion of the assembly comprised of insertions according to each repetitive element’s divergence from a consensus sequence for the repeat family to which it belongs. This analysis reconstructs the history and magnitude of repetitive element invasion and expansion in a genome. When comparing the repeat landscapes of the anole genomes, we found that *A. carolinensis* contained a much higher proportion of transposable element insertions with ≤10% divergence from their family consensus (Fig. 3). This was for DNA transposons (Kruskal Wallis test; *P* = 0.0005), LTR retrotransposons (*P* = 8.09e-07), and LINEs (*P* = 0.0073), but not SINEs. This is consistent with previous analyses of these and other species^6^ and suggests that while the transposable element landscape of the *A. sagrei* genome includes more DNA transposons and LINEs than *A. carolinensis*, this discrepancy is driven by a much larger proportion of the genome comprised of ancient insertions (those with Kimura 2-parameter divergence in *A. sagrei* of 10% or greater). In contrast, the transposable element landscape of the *A. carolinensis* genome is dominated by recent inserts, which is indicative of recent activity.

Previously, inferring which anole lineage (*sagrei* or *carolinensis*) experienced changes resulting in the differences observed was not possible. However, the recent publication of the *Sceloporus undulatus* genome^71^ and its TE landscape’s similarity to *A. sagrei*, suggest that the lack of older insertions in *A. carolinensis* is a unique characteristic to that species that distinguishes it from other Pleurodont Iguanians which have a larger proportion of old TE insertions (Supplementary Data 5-6). Analyses including other *Anolis* genomes have similarly found the *A. carolinensis* TE landscape to be distinct^6,73^. While we are not able to definitively explain why *A. carolinensis* might have a different repetitive element landscape than other anoles and Iguanians generally, there is one available hypothesis that might explain this observation. Demographically, *A. carolinensis* has experienced two genetic bottlenecks in its evolutionary history - once from a migration event out of Cuba, and a second migration event out of Florida^74–77^. The resulting reduction in genetic diversity associated with serial bottlenecks could be responsible for the shift in TE distribution by fixing segregating genetic variation for a reduced complement of old TE insertions.

### Synteny analysis of the X chromosome

A growing body of evidence suggests that fusions between autosomes and sex chromosomes are common across anoles^78,79^. The chromosomes that result from such fusions, called neo-sex chromosomes, have been used to study multiple evolutionary processes, such as chromosomal degeneration^80^ (or lack thereof) and dosage compensation^81^. *Anolis sagrei* has an XY sex-determination system with sex chromosomes that are substantially larger than those from *A. carolinensis*^67,78^. As Iguanian lizards (except for Basilisks) share a highly conserved core of X-linked genes^82^, the enlarged sex chromosomes in *A. sagrei* have been hypothesized to be the product of three independent fusions of autosomes to conserved iguanian X and Y sex chromosomes^60,67^. By aligning *A. sagrei* short reads from chromosomal flow sorting to the reference *A. carolinensis* genome, Giovannotti and colleagues^67^ showed that the 7th largest chromosome in the *A. sagrei* karyotype was the product of a series of chromosomal fusion events that occurred in the *A. sagrei* lineage. Ancestral chromosomes homologous to *A. carolinensis*’ chromosomes 9 and 12 fused to chromosome 13, the X chromosome of *A. carolinensis* (henceforth’ancient X’). Soon after, Kichigin and colleagues found that the chromosome corresponding to *A. carolinensis* chromosome 18 had also fused to the ancient X in the *A. sagrei* lineage^61^. These authors hypothesized that the neo-sex chromosome in *A. sagrei* resulted from three fusion events: chromosomes 12 and ancient X would have fused independently from chromosomes 9 and 18, and these two pairs of fused chromosomes then fused together to create the current *A. sagrei* XY system. Kichigan further proposed a synteny hypothesis for the *A. sagrei* neo-sex chromosomes in which the ancient X and chromosome 18 would be at the extremes of the neo-X chromosome, while chromosomes 12 and 9 would be in its center^61^.

**Fig. 4:**
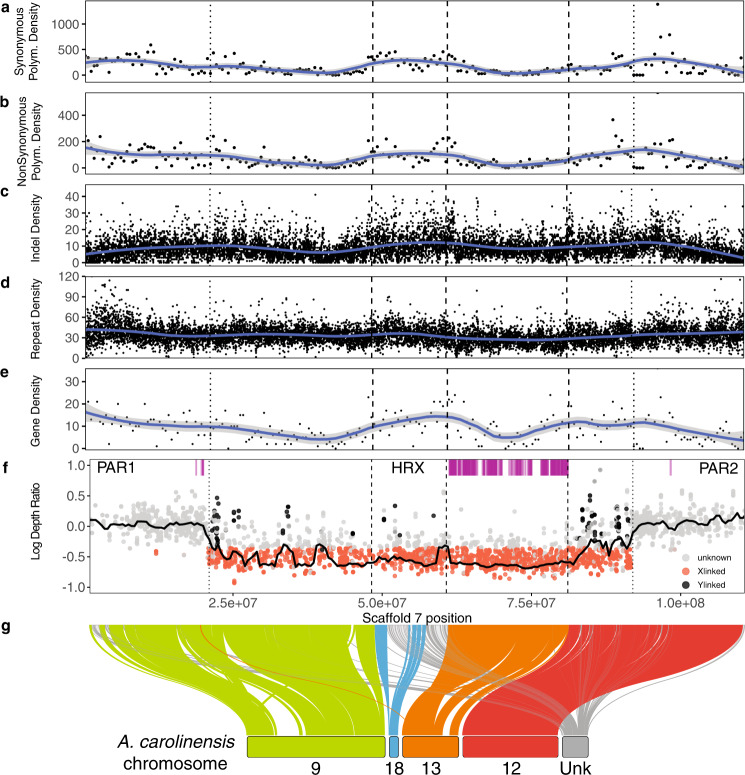
Identification and analysis of the X chromosome. **a**–**e** sliding window plots of element density with LOESS smoothed lines (span = 0.25) of **a**) synonymous SNPs per 500 kb, **b**) nonsynonymous SNPs per 500 kb, **c**) indels per 10 kb. **d** Repetitive elements per 10 kb, and **e**) Genes per 500 kb. **f**) Scaffold 7 male/female depth ratio (log-transformed). The black horizontal line summarizes depth ratio using a sliding window analysis (2 Mb windows, 500 kb step size). X-linked SNPs as those that are outliers for low sequencing depth ratio and show male heterozygosity equal to or lower than female heterozygosity. Y-linked SNPs correspond to significant sex GWA hits. Magenta ticks indicate the annotated location of *A. sagrei* homologs of X-linked genes in *A. carolinensis*. **g** Syntenic relationship between *A. sagrei* scaffold 7 and *A. carolinensis* chromosomes. For all panels, dashed lines represent the boundaries between regions homologous to different A. carolinensis chromosomes and dotted lines mark the estimated boundaries of pseudoautosomal regions (PAR1 and PAR2) and the putative Hemizygous Region of the X (HRX). Underlying data appear in Supplementary Data 8, 10-13.

Analyses of read depth, heterozygosity, and genome-wide association all indicate that AnoSag2.1 scaffold 7 is the X chromosome in this species (see *Sex Chromosome Identification*

below). Using SatsumaSynteny, we aligned the *A. carolinensis* and *A. sagrei* genomes and confirmed previously published predictions^61,67^ that the X chromosome in *A. sagrei* is the product of fusions between chromosomes homologous to 9, 12 and 18 from the *A. carolinensis* assembly and the ancient X. Given the level of contiguity of the AnoSag2.1 scaffold 7, our results present a clear synteny prediction for not only the order of the *A. carolinensis* chromosomes in scaffold 7, but also for the linkage groups that make up the ancient X in *A. carolinensis* (Fig. 4g). We found extensive overlap between the list of scaffolds identified in our synteny analyses and those obtained using short-read data from chromosomal flow sorting^67^. Furthermore, our data corroborated previous results based on dosage compensation, qPCR of X-linked genes and flow sorting in *A. carolinensis* that identified 8 additional scaffolds as X-linked in the original *A. carolinensis* assembly^12,61,83,84^.

Although our synteny data confirmed the identity of the ancestral chromosomes that fused to the ancient X to make up *A. sagrei*’s neo-sex chromosomes, our results do not support previous predictions of how these chromosomes are ordered within the *A. sagrei* neo-X chromosome. Our data suggest that chromosomes 18 and the ancient X are fused together at the center rather than at the extremes of scaffold 7 (Fig. 4g). In addition, only minor rearrangements are evident within formerly autosomal chromosomes, which suggests high levels of synteny within chromosomes despite their fusion to each other and the ancient X (Fig. 4).

We found that all nine linkage groups that had been previously assigned to the ancient X aligned to a 20.35 Mb stretch near the center of scaffold 7 (Supplementary Data 7). Two *A. carolinensis* chromosomes - GL343282.1 and chromosome 13 (the X chromosome) are the only linkage groups with regions aligning to multiple locations on *A. sagrei* scaffold 7. Chromosome 13’s other alignment is relatively short (˜1.6 Mb) and is located in one of two hypothesized pseudoautosomal regions (see below); GL343282.1’s other hit is also within the boundaries of the region homologous to the ancient X (see Supplementary Data 8). In addition to partially corroborating Kichigin and colleagues^61^ hypothesis and predicting the orientation of the ancestral autosomes along *A. sagrei*’s neo-sex chromosome system (including the ancient X), our syntenic alignment also identified an additional 142 linkage groups from the *A. carolinensis* assembly as being X-linked in *A. sagrei*’s scaffold 7 (Supplementary Data 8).

Our results, therefore, corroborate the hypothesis that the XY system in *A. sagrei* is composed of neo-sex chromosomes that originated through the fusion of chromosomes homologous to chromosomes 9, 12, 18 and the X in the *A. carolinensis* karyotype^61^. Furthermore, the high contiguity of scaffold 7 led us to hypothesize a new arrangement of these formerly autosomal chromosomes in the *A. sagrei* neo-X chromosome.

### Population genomic identification of the X chromosome

Previous studies have indicated that *A. sagrei* has a male heterogametic sex chromosome system^78,79,85^. Sex determination is genetic; however, females have been shown to preferentially use X or Y bearing sperm to fertilize eggs to skew sex ratios^53,54^. The sex chromosomes of this species are thought to be represented either by microchromosomes^85,86^ or by macrochromosomes^67^. Our synteny-based analyses (above) suggest that scaffold 7 is the X chromosome in *A. sagrei*. To independently verify which of the chromosomes in the *A. sagrei* genome are sex-linked, we used double-digest restriction site associated (ddRAD) data for 50 males and 50 females drawn from 16 populations distributed across the native and introduced ranges of *A. sagrei* (Supplementary Data 9). This method has previously been shown to perform well for sex chromosome identification in anoles^87^ and other taxa^88^.

After quality filtering, we retained an average of 2.3 M read pairs per sample, with no difference observed for males and females (*P* = 0.81; Wilcoxon rank sum test). The GWAS analysis performed using the final 120,967 filtered SNP set identified 204 markers distributed on scaffolds 1, 2, 3, 5, 6, and 7 as significantly associated with sex (Supplementary Figure 1a). Of these, the majority (i.e., 190 SNPs; 93.1%) were clustered on scaffold 7, where we also identified the strongest associations (Supplementary Figures 1a,b). Most (96%) significant GWAS hits showed an excess of heterozygosity in males relative to females (Supplementary Figures 1c,d), as expected if they are linked with the Y chromosome. Compared to the significant associations on scaffold 7, those occurring on scaffolds 1-6 showed an excess of sequencing coverage in males relative to females (Supplementary Figures 1c, d, e). Therefore, a reasonable interpretation is that these SNPs correspond to regions of the genome that have been duplicated between the autosomes and the Y chromosome.

Analysis of sequencing depth further supported our interpretation that scaffold 7 is the sex chromosome in *A. sagrei*. Specifically, scaffold 7 contains 89.6% of the genomic outliers with lower coverage in males compared to females (Fig. 4g). This result is consistent with heterogamety in *A sagrei*, and with X-linkage of sequencing depth outliers. X-linked SNPs are clustered along a 71 Mb region on scaffold 7, which also contains the Y-linked SNPs identified by GWAS (Supplementary Figure 1b).

Collectively, these results indicate the 71 Mb region on scaffold 7 corresponds to the putative Hemizygous Region of the X chromosome (HRX) in *A. sagrei*. The 21 Mb to the left of the HRX, and the 19 Mb to the right of the HRX mostly contain markers with even coverage between males and females. We infer that these correspond to recombining pseudoautosomal regions (PAR1 and PAR2; Fig. 4f). These pseudoautosomal region (PAR) boundaries appear to have evolved *de novo* since the divergence of *A. sagrei* and *A. carolinensis* as the entire ancestral X chromosome (and therefore the ancestral PARs) lies within the HRX in *A. sagrei*. This suggests that Y chromosome degradation encompasses all regions homologous to *A. carolinensis* chromosomes 13 (the X) and 18 and much of chromosomes 9 and 12. The PARs of the *A. sagrei* X chromosome are now composed of portions of two former autosomes, *A. carolinensis* chromosomes 9 and 12. A similar – but far more ancient – event has been hypothesized to have occurred in eutherian mammals where one of the two PARs present in these species arose after the divergence of monotremes and placental mammals 80–130 million years ago^89^. Two recent studies place the divergence of *A. sagrei* and *A. carolinensis* at less than 50 million years ago^90,91^ suggesting that *A. sagrei* has evolved two new PARs in roughly half the time placental mammals evolved one.

As discussed above, we detected broad sequence homology between *A. sagrei* scaffold 7 and nine X-linked *A. carolinensis* scaffolds which together contain 272 gene models in the NCBI RefSeq^92^ *A. carolinensis* annotation (release 102). We found 231 orthologous gene models in our annotation of *A. sagrei*. The vast majority (225; 97%) of these appear on scaffold 7 in AnoSag2.1. Only five genes are annotated to occur on other scaffolds (*psmb8* and *tap2* – scaffold 2, *iscu* – scaffold 6, and *iqcd* – scaffold 9, and *ptges2* – scaffold 12). Most of these genes occur exclusively on scaffold 7, however, 17 genes have paralogs occurring both on scaffold 7 and another scaffold. Of the 225 genes on scaffold 7, all of them have at least one copy within the region homologous to the *A. carolinensis* X, chromosome 13 (Fig. 4f). Duplicate copies of two genes also occur elsewhere on scaffold 7. A single copy of *dnah10* is present in PAR1 and four copies of *cmklr1* occur in PAR1 and one copy in PAR2 (Supplementary Data 10). In mammals and flies the duplication or movement of genes to regions outside the HRX have been observed and are hypothesized to be associated with either dosage compensation or male-specific function. However, we are unable to find support for either hypothesis for these two genes.

*Anolis carolinensis* is the only squamate genome with anchored sex chromosomes identified cytogenetically^22^. As such, it is commonly used for comparative analysis of sex chromosome evolution. However, we and others have shown that the *A. carolinensis* scaffold identified as the X (LgB, the 13^th^ largest scaffold in the AnoCar2.0 assembly) is missing large portions of X-linked sequence^61,67^. While multiple authors have improved our understanding of what genomic regions are X-linked in *A. carolinensis*^12,61,67,83,84^, they have not done so via de novo assembly of the X chromosome. As a result, our scaffold 7 is an ideal candidate to represent squamates for comparative analyses of sex chromosomes due to the relatively close relationship of the anchored *A. carolinensis* X and *A. sagrei* X chromosomes and the previous observation that anole sex chromosomes are derived from a single ancestral pair^78,79^. This allows researchers to use our scaffold 7 as a starting point for comparative analysis of sex chromosomes within reptiles and beyond.

### X-autosome fusion

The Y chromosome of *A. sagrei* is roughly two-thirds the size of the X^67^. This reduction is likely via the process of Y chromosome degeneration^60,93^. Under this process, formerly homologous regions in the Hemizygous Region of the X chromosome (HRX) of the X and Y diverge through mutational accumulation and deletions on the Y. The HRX is expected to evolve under different evolutionary pressures than those on autosomes or within pseudoautosomal regions on the sex chromosomes because, when they occur in males, these loci are effectively haploid. Recessive deleterious genetic variants such as indels, non-synonymous mutations, or repetitive element insertions are thus exposed to purifying natural selection in males and are therefore more likely to be purged from a population^94^. Similarly, the hemizygosity of the X chromosome may result in more efficient positive natural selection^95,96^. Our highly contiguous assembly of the *A. sagrei* neo-sex chromosome is composed of ancient sex-linked sequences as well as more recently recruited former autosomes, we might expect variation in the density of variants among these regions, reflecting differences in the time they have been X-linked. Just such a phenomenon has been observed in the neo-X of *Drosophila miranda* where formerly autosomal portions of the X chromosome have reduced synonymous polymorphism due to repeated selective sweeps^97^. Indeed, our data suggest some gametologs on the X and Y have sufficiently diverged to allow detection of X- and Y-linked sequences in the HRX of *A. sagrei* (Fig. 4f). However, the mapping of male-linked sequences to regions homologous to *A. carolinensis* chromosomes 9, 12, and 18 but not the X (chromosome 13) reveals, unsurprisingly, that X-Y divergence is more substantial on the portion of the X chromosome that has been sex-linked the longest. We also observed differences in the density of indels, repetitive elements, genes, and synonymous and nonsynonymous polymorphisms among the sub-compartments of the *A. sagrei* X chromosome; regions homologous to the ancient X have a lower density of each of these features than regions homologous to *A. carolinensis* autosomes (Fig. 4a-e, Supplementary Data 11-13, Supplementary Figure 2). Future analyses, using population genetic data in contrast to the pooled sequencing performed here, would allow more detailed evaluation of the evolutionary dynamics at play on the *A. sagrei* X chromosome.

## Conclusions

We report a, high-quality genome assembly of the brown anole, *Anolis sagrei*. Our analyses of this genome have revealed insights into the lineage-specific accumulation of repetitive elements and the complex evolution of anole sex chromosomes, including multiple bouts of autosome-sex chromosome fusion. The highly contiguous nature of our assembly and its substantial completeness presents a community resource that will enable future and on-going work in this emerging model organism. The assembly and accompanying annotation of genes and genetic variation we report here make possible a wide array of analyses such as genetic mapping of traits^23,98^ and functional genetics. Finally, the assembly serves as a launching point for future work probing the genome of this diverse species, including the assembly of the Y chromosome and population-scale analysis of structural evolution.

## Methods

### Chosen animal

A single female *Anolis sagrei ordinatus* was chosen for sequencing. This animal was collected from the Conception Island Bank in the Eastern Bahamas. Mitochondrial sequencing from across the range of the species had previously revealed this population to have the lowest levels of nucleotide polymorphism^99^ and was therefore best suited for *de novo* genome assembly. After humane euthanasia using Sodium Pentobarbital, we excised and flash froze muscle and liver tissue in liquid nitrogen. Flash frozen tissues were subsequently stored at −80 °C. The carcass was preserved in ethanol and accessioned into the Herpetology collection of the Museum of Comparative Zoology (specimen number Geneva1000, MCZ R-198163). All animal work was performed under Harvard Institutional Animal Care and Use Committee Protocol 26-11. Research, collection, and export permissions were granted by the Bahamas Environment, Science and Technology Commission, the Bahamas Ministry of Agriculture and Marine Resources, and the Bahamas National Trust.

### Sequencing

High Molecular Weight DNA was extracted from muscle and liver tissues using a Qiagen genomic tip kit. Two whole genome shotgun sequencing libraries were prepared using a TruSeq v3 DNA PCR-free library preparation kit with a 450 bp insert between pairs.

Two Chicago libraries and three Dovetail HiC libraries were prepared following previously published protocols^64,100^. For Chicago libraries, ~500 ng of DNA was reconstituted into chromatin in vitro and then fixed in formaldehyde. For HiC libraries chromatin was first fixed in place with formaldehyde in the nucleus and then extracted. The remaining steps for both protocols were identical. Fixed chromatin was digested with DpnII, creating 5’ overhangs which were filled with biotinylated nucleotides followed by ligation of free blunt ends. Crosslinks were then reversed, and the DNA purified from protein. Purified DNA was treated to remove biotin that was not internal to ligated fragments. The DNA was then sheared to an average fragment size of 350 bp and used to generate sequencing libraries using NEBNext Ultra enzymes and Illumina-compatible adapters. Biotin-containing fragments were isolated using streptavidin beads before PCR enrichment of each library.

The two whole genome shotgun (WGS) libraries were multiplexed and sequenced across two sequencing lanes. The two Chicago and three HiC libraries were multiplexed and sequenced across two additional lanes. All libraries were sequenced as paired end 150 bp reads on the Illumina HiSeqX platform. A summary of the data generated from all sequencing approaches can be found in Supplementary Data 14. Raw sequence reads are accessioned on the NCBI SRA (BioProject ID: PRJNA783271).

### de novo assembly

We processed raw Illumina WGS reads using trimmomatic v.0.36^101^. We used ILLUMINACLIP to remove TruSeq3 v2 sequencing adapters. We then removed any nucleotides with quality scores less than 20 from the leading and trailing ends of each read. Next, reads were truncated from the ends if sliding windows of 13 bp have an average quality below 20. Finally, we retained only reads longer than 23 nucleotides. For trimmed reads less than 23 bp we removed both that read and its paired read. We retained 896 million read pairs after filtering. These reads were used as input for *de novo* assembly using meraculous v2.2.2.5^63^ with the following parameters (diploid mode - diploid nonredundant haplotigs, kmer size 73, minimum kmer frequency 8).

### Scaffolding

We used the initial *de novo* assembly, Chicago library reads, and Dovetail HiC library reads as input data for HiRise v2.1.6-072ca03871cc, a software pipeline designed specifically for using proximity ligation data to scaffold genome assemblies^64^. We performed an iterative process of scaffolding. First, Chicago library sequences were aligned to the *de novo* input assembly from meraculous using a modified SNAP read mapper (http://snap.cs.berkeley.edu). The mapped separation of Chicago read pairs within draft scaffolds were analyzed by HiRise to produce a likelihood model for genomic distance between read pairs, and the model was used to identify and break putative misjoins, to score prospective joins, and make joins above a threshold. After aligning and scaffolding using Chicago data, HiC library sequences were aligned and used for scaffolding following the same method above but with the Chicago-scaffolded assembly as input.

### AnoSag1.0

Using the Chicago-scaffolded assembly as input we used abyss-sealer v2.02^102^ with options “-v -j32 -s100G -k96 -k80 -k64 -k48 -P 50 -o run20 -B5000” to close 18.6% of gaps in the assembly, substituting 9 Mb of ambiguous sequence with determined bases and increased N50 by 1.2 Mb. Gap-filled scaffolds were ordered and named sequentially according to descending size.

The total length of this assembly (1.6 Gb) was substantially smaller than the *Anolis carolinensis* assembly or cytological estimates of *A. sagrei* (~1.9 Gb), so we performed an additional round of improvement to identify missing sequences. Using bwa-mem v0.17^103^, we mapped the Illumina WGS PE reads generated in the project to the v1.0 assembly and extracted all unmapped reads. Approximately 2.5% of all read pairs either did not map or only a single read mapped. We then performed a *de novo* assembly using ABySS v2.02^104^ using the unmapped paired and unpaired reads as input and a kmer size of 96, generating ~29 K contigs. BLASTN v2.7.1^105^ annotation of these contigs against the NCBI nr database revealed that about half had a highest match to saurian sequences (14,360; of which 10,975 mapped to an *Anolis* accession). We reserved all contigs mapping to saurians and discarded all other contigs to avoid contaminant and metagenomic sequences. These contigs were appended to the final sagrei assembly and are numbered in descending size. The version 1.0 assembly, including both gap-filled scaffolds and newly assembled and filtered contigs was composed of 28,096 elements (scaffolds plus contigs) totaling 1.62 Gb in length.

### AnoSag2.0

During quality control checks of the v1.0 assembly, we discovered an issue that led us to further refine the assembly. Specifically, during spot-checking of the annotation of deeply conserved developmental genes, we discovered that while our assembly placed exons in the same order as other vertebrate genomes the orientation of exons within genes varied substantially. This seems to be caused by the inability of scaffolding software to determine the orientation of some contigs while performing scaffolding using Chicago and HiC data^106^. To correct this issue, we generated additional long-read Pacific Biosciences data, broke the assembly back into contigs, and re-scaffolded.

### High molecular weight DNA isolation and sequencing

High-molecular-weight genomic DNA was extracted from the muscle tissue of the same female *A. sagrei ordinatus* used for all previous sequencing. Frozen muscle tissue was homogenized with a pestle in freshly made lysis buffer (0.1 mM Tris, 1% Polyvinylpyrrolidone 40, 1% Sodium metabisulfite, 500 mM NaCl, 50 mM EDTA, 1.25% SDS, pH 8.0) and incubated with proteinase K at 55 °C for 50 minutes prior to RNase A treatment at room temperature for 10 minutes^107^. Next, one-third volume of 5 M potassium acetate was added, and the solution was incubated at 4 °C for 5 minutes and pelleted by centrifugation. The DNA in the supernatant was bound to SPRSelect beads (Beckman Coulter Life Sciences), washed and eluted in elution buffer (10 mM Tris, pH 8.0) according to the manufacturer’s instructions. Throughout the extraction process, the solutions were manipulated gently to minimize the shearing of DNA.

### PacBio sequencing

A SMRTbell library was constructed using the SMRTbell Template Prep Kit 1.0 (Pacific Biosciences) and sequenced on the Sequel I platform using Sequel Sequencing Kit 2.1 (Pacific Biosciences, Sequel SMRT Cell 1 M v2). Two sequencing runs generated a total of 1,257,251 reads, with an average size of 18 kb. Raw sequence reads have been accessioned on the NCBI SRA (BioProject ID: PRJNA783271).

### PacBio contig extension and bridging

The Illumina short read data generated for the initial *de novo* assembly (see above) were used to correct errors in PacBio long reads using Proovread v2.14.1^108^. As a trade-off between run-time and accuracy, 40x short read coverage was used during error correction. The resulting untrimmed error-corrected PacBio reads were subjected to additional hybrid error correction with FMLRC^109^ before being used to extend and bridge the original contigs from the AnoSag1.0 assembly. The AnoSag1.0 genome assembly was reverted to contigs by breaking scaffolds at any gap of 100 bp or more. The resulting contigs were extended and bridged using error-corrected PacBio reads using SSPACE-LongRead scaffolder v1.1^110^. Redundant contigs were removed using fasta2homozygous.py, a python script from Redundans v0.14a^111^. BUSCO assessments using the vertebrata dataset were performed before and after the removal of redundant contigs to ensure that removing redundant contigs did not change the completeness of the genome assembly. Gaps in contigs were closed by LR Gapcloser^112^. Contigs were then re-scaffolded through two iterations of the HiRise pipeline using previously prepared Chicago and Hi-C libraries as described above to generate the version 2 *Anolis sagrei* genome assembly – AnoSag2.0. All programs were run using the recommended default settings.

### AnoSag2.1

We generated a link density histogram paired reads from our HiC library using Juicer v1.6^66^. Visualizing these data in Juicebox v1.11.08^113^ revealed the second largest scaffold was in fact an intercalated fusion of two large scaffolds (Supplementary Figure 3). Through inspection of HiC link data as well as read mapping of Illumina short-read, RNA-Seq, and PacBio data, we identified three breakpoints on the AnoSag2.0 scaffold_2 (positions 1-206,031,901; 206,031,902-209,142,770; 209,142,7701-210,003,944; and 210,003,945-342,856,123) resulting in 4 fragments. We split the scaffolds at these locations and rejoined the first fragment to the third, and the second fragment to the fourth according to evidence from HiC link mapping. This resulted in the formation of two new scaffolds – the fifth and sixth largest in the new assembly. We sorted and renamed scaffolds by size to create the final AnoSag2.1 assembly reported here. We are making earlier assemblies (AnoSag1.0 and AnoSag2.0) publicly available because earlier research has been performed and published using those preliminary assemblies.

### Mitochondrial genome assembly

The mitochondrial genome was absent from the AnoSag2.1 assembly. To assemble the mitochondrial genome, we first subsampled 1 million trimmomatic filtered and trimmed Illumina read pairs. These reads were used as input for a circular *de novo* assembly in Geneious v11.1.5 (https://www.geneious.com). Separately, we extracted the largest subread (17.2 kb) from our error-corrected PacBio dataset. These two sequences were identical at the nucleotide level where they overlapped, but each contained regions absent in the other. To complete the mtGenome assembly we created a consensus of these two sequences and then aligned both PacBio and Illumina reads to that consensus to confirm reads from both platforms aligned to all regions. We annotated the mitochondrial genome assembly using the MITOS webserver^114^.

### Chromosome size analysis

Using a recently published, high-resolution *Anolis sagrei* karyotype (Fig. 1c from Giovannotti and colleages^67^) we measured the size of each chromosome as it appeared in that figure. For each chromosome, we calculated the fraction of the total karyotype occupied by that chromosome for an XX individual and multiplied that fraction by the total size of the AnoSag2.1 assembly to generate an estimate of nucleotide content. These estimates were then compared against the number of nucleotides in each size-sorted AnoSag2.1 scaffold (Supplementary Data 15). We calculated the correlation between scaffold size and estimated chromosome size via linear regression using the lm function in R v3.6^115^.

### Repetitive element content

To estimate the repetitive landscape of the *Anolis sagrei* genome, we modeled repeats *de novo* on the assembly using RepeatModeler v1.08^116^ and annotated the repeat consensus sequences using RepeatMasker v4.0.7^117^. To understand the age distribution of transposable elements in each genome, we used the divergence of an insert from its family consensus as a proxy for its age. We generated alignments for each repeat family and calculated the Kimura-2 parameter divergence from consensus (correcting for CpG sites) using the calcDivergenceFromAlign.pl RepeatMasker tool. We compared the repetitive profiles of *A. sagrei* and *A. carolinensis* through a parallel analysis, running RepeatModeler and RepeatMasker with the AnoCar2.0 assembly^22^.

### Gene model annotation

For gene structure annotation of AnoSag2.1, we ran Braker v2.0.5^72^ using RNA-Seq data and amino acid sequences of closely related species. In brief, we used RepeatModeler v1.0.11^116^ to construct an *Anolis sagrei* repeat library, which was subsequently used by RepeatMasker v1.0.11^117^ to mask repeats in the genome. We used protein sequences of *A. carolinensis* and *A. punctatus* obtained from NCBI RefSeq to query our reference sequence for homologous proteins. Composite RNA-seq data were prepared by combining eight paired-end RNA-Seq libraries consisting of two libraries from a forelimb and a hindlimb at embryonic stage 7^118^, three libraries from brain, liver, and skin tissue of an adult female^119^ (SRA accession number: DRA004457), and three new libraries from central, nasal, and temporal regions of eye retina. We generated these eye RNA-seq datasets from tissues from Sanger St 16.5 embryos laid from wild-caught *A. sagrei* parents from Orlando, FL. We collected tissues from the nasal, central, and temporal posterior regions of the eye, and pooled together samples from 3 embryos (of mixed sex). We isolated total RNA using the mirVana RNA Isolation Kit (ThermoFisher Scientific). We constructed libraries using the TruSeq Stranded mRNA Sample Prep Kit for Illumina and sequenced on an Illumina NextSeq 500 platform. All combined RNA-seq read data were aligned to AnoSag2.1 using TopHat v2.1.1^120^ with the option --b2-very-sensitive. The Braker gene prediction pipeline was run with the options “--softmasking --prg=gth --gth2traingenes”.

CD-HIT v4.6.8^121,122^ was used with default parameters to remove redundant gene models from Braker’s output. Using the protein sequences of non-redundant gene models from CD-HIT as a query, BLASTP v2.7.1^105^ searches were performed against the non-redundant RefSeq protein database. Gene models with unique protein matches and e-value less than 1e-3 were kept. When more than one gene model had blast hits with the same protein, the gene model with the best score was kept. In addition, we retained gene models that lacked a BLAST hit if they either 1) contained 3 or more exons or 2) had more than 50 RNA-seq reads per 15 million mapped reads and did not overlap with those from the non-redundant CD-HIT gene models already retained. Gene models from these processes were combined to generate a final non-redundant gene set. We removed gene models that consisted of highly repetitive sequences with no available RNA-seq supportive evidence. To systematically detect instances where genes were inappropriately split into two or more gene models, we parsed BLAST results to identify all adjacent gene models that matched to the same protein. We visually examined aligned RNA-seq reads to these gene models individually in IGV v2.11.9^123^ to determine whether there was evidence of splicing between adjacent gene models, and adjacent gene models were merged if there was evidence of splicing. To assign gene names to gene models, we used BLASTP on translated protein sequences of gene models against NCBI vertebrata RefSeq protein database and then converted RefSeq protein accession (as input) into gene symbol (as output) using db2db in bioDBnet (https://biodbnet-abcc.ncifcrf.gov/db/db2db.php).

### SNP/indels genotyping

We performed sequence variant calling with composite shotgun data by combining 75 bp single-end Illumina reads from 5 genomic libraries originally generated as control data for *A. sagrei* ChIP-seq experiments. Each genomic library was created from a pool of embryos produced by a colony of wild-caught *A. sagrei* from Orlando, FL. The library details are as follows: library 1, 57 embryos, 46.6 million reads; library 2, 59 embryos, 42.1 million reads; library 3, 91 embryos, 16.6 million reads; library 4, 97 embryos, 104 million reads; library 5, 70 embryos, 25.3 million reads. Likewise, composite RNA-Seq data were generated by combining data from 27 RNA-Seq libraries from embryonic (forelimbs, hindlimbs, and retina) and adult tissues (brain, skin, and liver). Embryonic limb (GEO accession GSE128151) and adult tissue RNA-seq data (DDBJ Sequence Read Archive accession DRA004457) were previously published^118,119^. Anole eye RNA-seq datasets were generated from tissues from Sanger Stage 16.5 embryos laid from wild-caught *A. sagrei* parents from Orlando, FL. Tissues from the nasal, central, and temporal posterior regions of the eye were collected, and samples from 3 embryos (of mixed sex) were pooled together. Total RNA was isolated using the mirVana RNA Isolation Kit (ThermoFisher Scientific). Libraries were constructed with TruSeq Stranded mRNA Sample Prep Kit for Illumina and sequenced on the Illumina NextSeq 500 platform. Embryonic retina RNA-seq data were submitted to GEO (GEO accession GSE184570). The composite sequence data were aligned to the *A. sagrei* genome (AnoSag2.1) using BWA-mem v0.7.15^103^ with the default parameters. The resulting alignment files (SAM format) were merged and converted into sorted BAM file using SAMtools v1.6^124^ before duplicates were removed using Picard v2.16.0 (Broad Institute, 2019) MarkDuplicates with the options “MAX_FILE_HANDLES_FOR_READ_ENDS_MAP = 1000 REMOVE_DUPLICATES = true ASSUME_SORTED = true VALIDATION_STRINGENCY = LENIENT”. SAMtools mpileup was used to generate genotype likelihoods at each genomic position with coverage from the deduplicated BAM file. We used BCFtools v1.9^125^ with the options “--keep-alts --multiallelic-caller --variants-only” to call and filter sequence variants. We further filtered single nucleotide variants using VCFTools v0.1.15^126^ to have a minimum quality score of 25 and a minimum depth of 5 reads (commands “--minQ 25 --minDP 5”). Summaries of features per genomic window (indels, SNPs, genes, repetitive elements) were calculated using VCFTools and BEDTools v2.26^127^. The impact of single nucleotide variants was assessed using SNPeff v5.0^128^ using default settings.

### Analysis of X chromosome synteny

We used SatsumaSynteny v2.0^129^ to align scaffold 7 in the *A. sagrei* assembly to the *A. carolinensis* assembly version 2.0. Next, we used custom awk scripts to modify SatsumaSynteny’s default output to a bed format and used the Circos^130^ function bundlelinks to merge adjacent links together. We used bundlelinks’’strict’ flag, keeping only bundles that were within 1Mbp of each other and that were at least 1kbp in length. To make the linear synteny plot between *A. sagrei* scaffold 7 and the scaffolds in the *A. carolinensis* assembly, we used the R package RIdeogram^131^ in R v3.6^115^. For plotting purposes, we labeled scaffolds that aligned to scaffold 7 as belonging to either chromosome 9, 12, 18 or the ancient X in the *A. carolinensis* assembly using information from previous flow sorting and dosage compensation studies in *A. carolinensis*^12,61,67,83,84^.

We assessed the synteny degree between the *A. sagrei* and *A. carolinensis* genomes using Satsuma v3.1.0^129^, an alignment software devised to deal with large queries and references. Satsuma works as follows. First, it breaks the query and the reference sequences into 4096 bp chunks, by default, that overlap in one-quarter of their size. Then, it translates As, Cs, Ts and Gs into numeric signals that go through cross-correlation. Cross-correlation is calculated as a function of the displacement of one sequence’s signal relative to the other. It measures the similarity among two analog signals, the higher the cross-correlation value, the more bases match across the overlap and the stronger the signal. Next, Satsuma fine-tunes the alignment by keeping sequences that are at least 28 base-pairs long and have 45% matching. Then, Satsuma calculates an alignment probability model based on the aligned sequences length, identity, GC content and length, keeping alignments that have probability lower than 10^−5^ of being random noise. After Satsuma identifies it proceeds with dynamic programming to merge overlapping blocks into alignments with gaps. To reduce computational time, Satsuma implements a ‘paper-and-pencil game battleship’ approach, in which it queries the vicinity of the alignment for more hits.

### Sex chromosome identification

We prepared libraries following the standard ddRAD protocol^132^. Briefly, we used the *SphI* HF and *EcoRI* HF restriction endonucleases (New England Biolabs) to digest genomic DNA. After size selection, we retained fragments of 500–660 bp. Libraries were pair-end sequenced (150 bp read length) on an Illumina HiSeq 4000 (Illumina, San Diego, CA, USA). We included ddRAD data for 50 males and 50 females, sequenced as part of a larger related project investigating the population genomics of native and invasive *A. sagrei* populations^23^. These were obtained from 16 populations distributed across the native and introduced ranges of *A. sagrei* (Supplementary Data 9). In selecting samples, we aimed for a balanced representation of both sexes for most populations. Raw data are accessioned in the NCBI SRA database (BioProject ID: PRJNA737437).

Sequencing files were de-multiplexed using *ipyrad* v0.7.15^133^. We removed low-quality bases and Illumina adapters using Trimmomatic v0.36^101^. Cleaned reads were used for SNP calling within the dDocent v2.2.20 pipeline^134^. In dDocent, reads were aligned to the *A. sagrei* assembly using BWA v0.7.16a-r1181^135^ at default parameters. We then performed joint variant calling using the 100 *A. sagrei* genotypes along with 925 other conspecific genotypes sequenced as part of related projects, in Freebayes v1.0.2^136^. The genotype calls for the 100 samples used here were filtered using vcflib (https://github.com/vcflib/vcflib). We kept only biallelic SNPs with MAPQ scores > 20. For the remaining markers, we coded genotypes that were supported by fewer than four reads as missing data. We subsequently kept only SNPs with data in at least 70% of samples, and those with minor allele frequency larger than 5%.

To identify Y-linked genomic regions, we performed a genome-wide association study (GWAS) in PLINK v1.0.7^137^. Because *A. sagrei* is known to have a male heterogametic sex chromosome system^67,78,85^, SNPs associated with one sex should be in close linkage with the Y chromosome. Specifically, we expect these SNPs to represent differences that occur between X and Y gametologs. We coded sex as a binary case/control variable. Prior to the GWAS analysis, we imputed any remaining missing data in the filtered SNP set using BEAGLE v5.0 at default parameters^138^. For association testing, we used Fisher’s exact test and set the genome-wide significance threshold using the Bonferroni correction for multiple comparisons (0.05/total number of tested markers). Further confirmation of GWAS results was obtained by calculating the difference in heterozygosity between males and females (i.e. relative male heterozygosity), at each SNP. This is because in a male-heterogametic system we expect SNPs occurring between gametologs to show an excess of heterozygosity.

To identify X-specific genomic regions, we used the ratio of sequencing coverage between males and females. In species with heteromorphic sex chromosomes such as *A. sagrei*, this metric should be effective in distinguishing between the autosomes and the X chromosome^88^. A ratio close to 1 is expected for autosomes, while a ratio of 0.5 is expected for the X chromosome, due to hemizygosity of males. For both sequencing depth ratio and relative male heterozygosity, we identified upper and lower thresholds for categorizing SNPs as genomic outliers using the interquartile range (IQR; upper/lower quartile + /- 1.5 IQR). We then defined X-linked SNPs as those that are outliers for low sequencing depth ratio and show male heterozygosity equal to or lower than female heterozygosity. Y-linked SNPs were defined as those with significant sex GWA hits. Lastly, we used these SNP categories to identify the approximate boundaries of the PARs, by tallying the percent of sex-linked SNPs in 1 Mb windows along scaffold 7 (Supplementary Data 16).

### Statistics and reproducibility

The genome assembly reported here was derived from a single *Anolis sagrei* female. The source specimen has been accessioned into Herpetology collection of the Museum of Comparative Zoology (specimen number Geneva1000, MCZ R-198163). Our annotation pipeline made use of pooled RNA libraries consisting of 3-97 individuals. Population genomic and GWAS analyses were performed on a set of 100 individuals (50 males and 50 females). GWAS significance thresholds were adjusted using a Bonferroni correction for multiple comparisons (0.05/total number of tested markers).

## Supplementary information

Supplementary Information

Editorial Assessment Report

Description of Additional Supplementary Files

Supplementary Data 1 - 16

## Data Availability

The following resources are available at the Harvard Dataverse Repository, https://dataverse.harvard.edu/dataset.xhtml?persistentId=doi:10.7910/DVN/TTKBFU: the current AnoSag2.1 assembly (fasta format), annotation (gtf format), mtDNA assembly (fasta format), and AnoSag2.1 mtDNA annotation (gff format). This repository includes annotated SNP variants (vcf format), indel variants (vcf format), transcripts (fasta format), and proteins (fasta format) mapped to, or derived from, the AnoSag2.1 assembly. Also accessioned on Dataverse are previous, now deprecated, iterations of the *Anolis sagrei* genome: the AnoSag2.0 assembly (fasta format) and annotation (gff, deprecated annotation), as well as the AnoSag1.0 assembly (fasta format). Newly produced raw and processed data have been accessioned under NCBI BioProject PRJNA783271. The AnoSag2.1 assembly is accessioned under NCBI genome ID JANCLY000000000. Raw sequencing data are available on the NCBI Sequence Read Archive: Illumina short reads (SRR21197535 and SRR21197563), Chicago Proximity Ligation data (SRR20983434), HiC proximity ligation (SRR20983433), and PacBio long reads (SRR20045886). Accession data for RNAseq data used for annotation are as follows: Embryonic retina (GEO accession GSE184570); Embryonic limb (GEO accession GSE128151); and adult tissue RNA-seq data (DDBJ Sequence Read Archive accession DRA004457). Raw ddRAD data are accessioned in the NCBI SRA database (BioProject ID: PRJNA737437). Source data underlying main figures are presented in Supplementary Data 1 (Fig. 1), Supplementary Data 3 (Fig. 2d) and Supplementary Data 5-6 (Fig. 3).
