# Supplementary material for: Chromosome-scale genome assembly of the brown anole (Anolis sagrei), an emerging model species
Source: Commun Biol. 2022 Oct 25;5:1126. doi: 10.1038/s42003-022-04074-5 (PMC9596491; doi:10.1038/s42003-022-04074-5)
Supplement: Supplementary file 2 — Editorial Assessment Report [file 42003_2022_4074_MOESM2_ESM.pdf]

nature portfolio

GUIDED OPEN ACCESS

nature genetics | nature communications | communications biology

Contents of this report

1. **Manuscript details**: overview of your manuscript and the editorial team.
2. **Review synthesis**: summary of the reviewer reports provided by the editors.
3. **Editorial recommendation**: personalized evaluation and recommendation from all 3 journals.
4. **Annotated reviewer comments**: the referee reports with comments from the editors.
5. **Open research evaluation**: advice for adhering to best reproducibility practices.

About the editorial process

Because you selected the **Nature Portfolio Guided Open Access** option, your manuscript was assessed for suitability in three of our titles publishing high-quality work across the spectrum of genetics research: ***Nature Genetics***, ***Nature Communications***, **and *Communications Biology***. More information about Guided Open Access can be found here.

Collaborative editorial assessment

Your editorial team discussed the manuscript to determine its suitability for the Nature Portfolio Guided OA pilot. Our assessment of your manuscript takes into account several factors, including whether the work meets the **technical standard** of the Nature Portfolio and whether the findings are of **immediate significance** to the readership of at least one of the participating journals in the Nature Portfolio Guided Open Access genetics cluster.

Peer review

Experts were asked to evaluate the following aspects of your manuscript:

- **Novelty** in comparison to prior publications;
- **Likely audience** of researchers in terms of broad fields of study and size;
- **Potential impact** of the study on the immediate or wider research field;
- **Evidence** for the claims and whether additional experiments or analyses could feasibly strengthen the evidence;
- **Methodological detail** and whether the manuscript is reproducible as written;
- Appropriateness of the **literature review**.

Editorial evaluation of reviews

Your editorial team discussed the potential suitability of your manuscript for each of the participating journals. They then discussed the revisions necessary in order for the work to be published, keeping each journal's specific editorial criteria in mind.

Journals in the Nature portfolio will support authors wishing to transfer their reviews and (where reviewers agree) the reviewers' identities to journals outside of Springer Nature.
If you have any questions about review portability, please contact our editorial office at guidedoa@nature.com.

nature portfolio

GUIDED OPEN ACCESS

nature genetics | nature communications | communications biology

Manuscript details

Tracking number	Submission date	Decision date	Peer review type
GUIDEDOA-21-00343	Nov 27, 2021	Jan 28, 2022	Single-blind

Manuscript title
Chromosome-scale genome assembly of the brown anole (*Anolis sagrei*), an emerging model species

Preprint: https://www.biorxiv.org/content/10.1101/2021.09.28.462146v1

Author details

Anthony Geneva

Affiliation: Rutgers University

Editorial assessment team

**Primary editor**	**Michael Fletcher**<br>Home journal: ***Nature Genetics***<br>ORCID: **0000-0003-1589-7087**<br>Email: **michael.fletcher@nature.com**
**Other editors consulted**	**Rebecca Furlong**<br>Home journal: ***Nature Communications***<br>ORCID: **0000-0002-8451-6471**<br><br>**George Inglis**<br>Home journal: ***Communications Biology***<br>ORCID: **0000-0002-9069-5242**
**About your primary editor**	Michael Fletcher studied for his Ph.D. at Cancer Research UK's Cambridge Institute at the University of Cambridge in the laboratory of Bruce Ponder. His project used systems biology approaches to identify the functional mechanism by which FGFR2, a breast cancer risk locus identified using GWAS, exerts its effect. He then moved to Germany and was a postdoctoral fellow in the Molecular Genetics department of Peter Lichter at the German Cancer Research Center in Heidelberg, where he performed bioinformatics analysis to help characterize the epigenomic and master regulator landscapes of adult glioblastoma. He joined the journal in 2020.

Editorial assessment and review synthesis

**Editor's summary and assessment**	This manuscript presents a reference genome assembly for the brown anole (*A. sagrei*), an emerging model organism notable for being the first lizard in which CRISPR was performed. This AnoSag2.1 reference is generated from short- and long-read DNA sequencing, along with Hi-C scaffolding, in line with the current best practice. Analysis of the genome and population genomics confirms the previously-proposed X chromosome origin via fusion of green anole autosomes and an ancient X.<br><br>Our editorial assessment pre-review was that this reference genome appears to be of high quality and that the assembly will be important for the anole/lizard community. It was unclear to us whether there would be broader appeal, as the biological findings presented seem likely to be of greatest interest to those studying these and related lizards. Therefore, we decided to send this to review, targeted at *Communications Biology*.
**Editorial synthesis of reviewer reports**	The two referees both sound appreciative of this work and the impact it will have in the field, saying that the genome is "an important contribution to reptile genomics" and that there is a "huge community" awaiting it. Neither referee raised any major technical concerns.<br><br>There were also some useful suggestions for improvement that we believe should be acted upon: most notably, Reviewer #1 suggests that the comparison with *A. carolinensis* could be expanded to other reptile genomes. We agree that this would be a useful extension of your analysis and would broaden the audience for your work.

Editorial recommendation

Nature Genetics<br><br>Revision not invited	While *Nature Genetics* appreciates the quality of this new reference genome and its usefulness for the field, the unclear broader impact means that a revision would not be invited.
Nature Communications<br><br>Revision not invited	*Nature Communications* recognises the value of this resource for reptile genomics, and the interest of the sex chromosome analysis, but significantly stronger followup analyses would be needed to make the work suitable for this journal.
Communications Biology<br><br>Minor revisions	*Communications Biology* would be interested in a revised manuscript that better compares the *A. sagrei* genome to other relevant reptiles (as outlined by Reviewer #1) and addresses the discussion points raised by Reviewer #2.

Next steps

**Editorial recommendation 1:**	Our top recommendation is to revise and resubmit your manuscript to *Communications Biology.* We feel the additional experiments required are reasonable to address within a 3-month timeline.
**Note**	As stated on the previous page *Nature Genetics* and *Nature Communications* are not inviting a revision at this time. Please keep in mind that the journal will not be able to consider any appeals of their decision through Guided Open Access.

Revision

To follow our recommendation, please upload the revised manuscript files using **the link provided in the decision letter**. Should you need assistance with our manuscript tracking system, please contact Adam Lipkin, our Nature Portfolio Guided OA support specialist, at guidedOA@nature.com.

Revision checklist

- ☐ Cover letter, stating to which journal you are submitting
- ☐ Revised manuscript
- ☐ Point-by-point response to reviews
- ☐ Updated Reporting Summary and Editorial Policy Checklist
- ☐ Supplementary materials (if applicable)

Submission elsewhere

If you choose not to follow our recommendations, you can still take the reviewer reports with you.

Option 1: Transfer to another Nature Portfolio journal
Springer Nature provides authors with the ability to transfer a manuscript within the Nature Portfolio, without the author having to upload the manuscript data again. To use this service, **please follow the transfer link provided in the decision letter.** If no link was provided, please contact guidedOA@nature.com.

> *Note that any decision to opt in to In Review at the original journal is not sent to the receiving journal on transfer. You can opt in to In Review at receiving journals that support this service by choosing to modify your manuscript on transfer.*

Option 2: Portable Peer Review option for submission to a journal outside of Nature Portfolio
If you choose to submit your revised manuscript to a journal at another publisher, we can share the reviews with another journal outside of the Nature Portfolio if requested. You will need to request that the receiving journal office contacts us at guidedOA@nature.com. We have included editorial guidance below in the reviewer reports and open research evaluation to aid in revising the manuscript for publication elsewhere.

Annotated reviewer reports

The editors have included some additional comments on specific points raised by the reviewers below, to clarify requirements for publication in the recommended journal(s). However, please note that all points should be addressed in a revision, even if an editor has not specifically commented on them.

Reviewer #1 information

**Expertise**	animal (reptile/lizard) genetics and genomics.
**Editor's comments**	This referee, with genomics expertise, acknowledges the importance and quality of the reference genome. We think that their suggestion for expanding the comparative analysis beyond *A. carolinensis* to other reptile species is a very interesting and potentially fruitful one.

Reviewer #1 comments

**Section**	**Annotated Reviewer Comments**
**Remarks to the Author: Overall significance**	This manuscript represents an important contribution to reptile genomics and the authors convincingly present a genome that will be a gold standard for several years. They sufficiently place their study in the context of previous studies and literature. I only have a few minor comments as detailed below.
**Remarks to the Author: Strength of the claims**	I do think that the conclusions are strong and that the authors did a good job merging their results with previous results, especially for the sex chromosome work (which seemed like the focus of the paper). One way I think the paper could be improved is with a more consistent comparison with other reptile genomes. The authors tout the *Anolis sagrei* genome as being the most complete and contiguous and do a great job at convincing the reader of this. However, I wonder if the authors can take this argument a bit further in some sections of the paper. For instance, what advantages does their assembly provide in studying sex chromosomes that were not possible in other reptile genomes beyond the main comparison with *A. carolinensis*? Another instance where I thought a comparison with other reptile genomes would be appreciated is in the discussion on annotation completeness, where the authors claim that looking at BUSCO analyses of the genome and of the exome (line 218). How does this comparison look in other reptile genomes with respect to the *Anolis sagrei* assembly?<br>**This point would be necessary for further consideration at *Communications Biology*.**<br><br>One other minor question I had is also related to the BUSCO analyses. In the paragraph starting on Line 191, I was wondering if the authors could very briefly discuss why genomes can be more contiguous but less complete, and vice versa, and why the *Anolis sagrei* genome has overcome these issues to be, essentially, the most contiguous AND complete reptile genome.
**Remarks to the Author:**	The methods are quite comprehensive for the most part; for instance, exact running parameters are given for some analyses. I would have liked to have had more detail

**Reproducibility**	on the manual annotation of genes (Line 638). What was meant by spot-checking a gene? What were the authors looking for, and how were the genes edited (or what is an example of how a gene model was edited)? What proportion of Braker gene models needed such editing?<br>**For the sake of reproducibility, please elaborate on these methods (there is no word limit for this section).**

Reviewer #2 information

**Expertise**	genetics and evolution.
**Editor's comments**	This referee was asked to bring a broader (i.e. non-genomics-specialist) view on the importance and impact of this novel genome. They sound highly enthusiastic and supportive of publication; notably, they say that the results are in line with past studies, giving confidence to the strength of such. Their requests for further changes are minor.

Reviewer #2 comments

Section	Annotated Reviewer Comments
**Remarks to the Author: Overall significance**	I read this paper with great interest. The genome for *A. sagrei* is indeed long overdue. The authors make a strong case for considering this species a relevant model and I agree that there are sufficient numbers of researchers using this species, its relatives, and other reptiles in general to make this an important genome. *A. sagrei* in particular has become a widely-studied "field model" in sub-disciplines ranging from population and quantitative genetics to behavior to physiology, to natural and sexual selection. As such, having its genome made available will serve as an important research tool to a huge community.
**Remarks to the Author: Impact**	With complete candour, I will say that I wonder whether at this point in the accumulating literature of new genomes, these types of papers are still of sufficient import that they should be published in *Nature* or its offspring journals. I think the results around the X-chromosome make this paper especially compelling and perhaps make the best case for publication in a high profile journal. Work by Cox and Calsbeek on the capacity for *A. sagrei* to control sex determination should be cited in this section (Science 2010 328: 92-94; Evolution 2008 62: 1137-1148).<br>**Concerns about novelty and impact prohibited further consideration by *Nature Genetics* and *Nature Communications*.**<br><br>The paper is well written and reports interesting results particularly those

	pertaining to the evolution of sex chromosomes in this group. I have very few comments on the methods or statistics in the paper as I am not technically savvy enough to critique these elements (and what I do know from this literature leaves me feeling comfortable that the data were produced and analysed correctly). I'm sure the other referees will chime in on these aspects. One area that I thought could use a bit more attention is the set of results shown in Fig3. The difference in transposable element activity in the two anole genomes is mentioned briefly but not tackled in a way that provides much insight. Why the difference in the two species? Their 50 million years of separation may be sufficient explanation but this paragraph left me wanting more in terms of discussion.<br>**Please address this discussion point for further consideration at *Communications Biology*.**
**Remarks to the Author: Strength of the claims**	The statement (line 103) that *A sagrei* is an emerging model for numerous fields seems like it should be simple to support with citations.<br><br>The legend to Fig 1. States that interest has grown over the last 40 years. Really it's only over about the last 18 or so….<br><br>Line 399 typo "degeneration"

Open research evaluation

General information

Guidelines for Transparency and Openness Promotion (TOP) in Journal Policies and Practices ("TOP Guidelines")

The recommendations and requests in the table below are aimed at bringing your manuscript in line with common community standards as exemplified by the TOP Guidelines. While every publisher and journal will implement these guidelines differently, the recommendations below are all consistent with the policies at Nature Portfolio. In most cases, these will align with TOP Guidelines Level 2.

FAIR Principles

The goal of the recommendations in the table below related to **data or code** availability is to promote the FAIR Guiding Principles for scientific data management and stewardship (*Scientific Data* **3**: 160018, 2016). The FAIR Principles are a set of guidelines for improving 4 important aspects of digital research objects: **F**indability, **A**ccessibility, **I**nteroperability and **R**eusability.

ORCID

ORCID is a non-profit organization that provides researchers with a unique digital identifier. These identifiers can be used by editors, funding agencies, publishers, and institutions to reliably identify individuals in the same way that ISBNs and DOIs identify books and articles. Thus the risk of confusing your identity with another researcher with the same name is eliminated. **The ORCID website** provides researchers with a page where your comprehensive research activity can be stored.

Springer Nature collaborates with the ORCID organization to ensure that your research contributions (as authors and peer reviewers) are correctly attributed to you. Learn more at https://www.springernature.com/gp/researchers/orcid

Data availability
**Data Availability Statement**
Many journals, including all Nature Portfolio journals, require a Data Availability Statement in the manuscript as a condition of publication. The Data Availability Statement should be as detailed as possible and include accession codes or other unique IDs for deposited data, information about where source data can be found, and specify any restrictions to data access that may apply. At a minimum, the statement should indicate that data are available upon request and explain how data access can be granted. If data access is not possible, the reasons for this must be made clear in the Data Availability Statement.<br><br>More information about the Nature Portfolio data availability policy can be found here:<br><br>Additional information about Data Availability Statements and Springer Nature's data policies are available here:
**Mandatory data deposition**
Most scientific journals, including all Nature Portfolio journals, require that any newly-generated DNA sequence data must be made publicly available before publication. There are some exceptions allowed for sensitive clinical data, but this should be discussed with the editor. All data must be deposited in a community-approved repository and accession codes/unique IDs must be included within the Data Availability Statement in the manuscript.<br><br>Examples of appropriate public repositories are listed below:<br>● GenBank<br>● Sequence Read Archive (WGS or WES data)<br>● The European Nucleotide Archive (ENA)<br><br>To follow best practice in the genomics research community, the full linked genotype-phenotype dataset for your genome-wide association study must be submitted to a community-endorsed, public repository. Please note that this is a prerequisite for publishing in a Nature Portfolio journal. Accession numbers must be provided in the paper within the Data Availability Statement. We recommend submission to the NCBI Sequence Read Archive (SRA): https://www.ncbi.nlm.nih.gov/sra<br>We also strongly encourage you to deposit full summary statistics to the NHGRI-EBI GWAS Catalog: https://www.ebi.ac.uk/gwas/<br><br>More information on mandatory data deposition policies at the Nature Portfolio can be found at: http://www.nature.com/authors/policies/availability.html#data<br><br>Please visit https://www.springernature.com/gp/authors/research-data-policy/repositories/12327124 for a list of approved repositories for various data types.

Please ensure that datasets deposited in public repositories are now publicly accessible, and that accession codes or DOI are provided in the "Data Availability" section. As long as these datasets are not public, we cannot proceed with the acceptance of your paper. For data that have been obtained from publicly available sources, please provide a URL and the specific data product name in the data availability statement. Data with a DOI should be further cited in the methods reference section.

Other data requests

In line with community standards regarding open research, Springer Nature strongly supports data sharing and believes that all datasets on which the conclusions of the paper rely should be available to readers. We encourage authors to ensure that their datasets are either deposited in publicly available repositories (where available and appropriate) or presented in the main manuscript or additional supporting files whenever possible.

To learn more about data sharing and recommended data repositories, please see: https://www.springernature.com/gp/authors/research-data-policy/repositories/12327124

Data citation

Please cite (within the main reference list) any datasets stored in external repositories that are mentioned within their manuscript. For previously published datasets, we ask that you cite both the related research article(s) and the datasets themselves. For more information on how to cite datasets in submitted manuscripts, please see our data availability statements and data citations policy:

Citing and referencing data in publications supports reproducible research, by increasing the transparency and provenance tracking of data generated or analysed during research. Citing data formally in reference lists also helps facilitate the tracking of data reuse and may help assign credit for individuals' contributions to research. A number of Springer Nature imprints are signatories of the Joint Declaration on Data Citation Principles, which stress the importance of data resources in scientific communication.

Code availability and citation

To adhere to community standards and promote transparency in research, any custom software or code should be made publicly available, ideally before publication so that referees can test the code and comment on it. Please include a statement under the heading "Code Availability", indicating whether and how the custom code/software reported in your study can be accessed, including any restrictions to access. This section should also include information on the versions of any software used, if relevant, and any specific variables or parameters used to generate, test, or process the current dataset. Code availability statements should be provided as a separate section after the Data Availability section.

Upon publication, Nature Portfolio journals consider it best practice to release custom computer code in a way that allows readers to repeat the published results. Code should be deposited in a DOI-minting repository such as Zenodo, Gigantum or Code Ocean and cited in the reference list following the guidelines described in our policy pages (see link below). Authors are encouraged to manage

subsequent code versions and to use a license approved by the open source initiative. Full details about how the code can be accessed and any restrictions must be described in the Code Availability statement. See here for more information about Nature Portfolio's code availability policies:

We also provide a Code and Software submission checklist that you may find useful:
https://www.nature.com/documents/nr-software-policy.pdf

Please note: because of advanced features used in this form, you must use Adobe Reader to open the document and complete it.

Ethics

We believe that authors, peer reviewers and editors should be required to disclose any competing interests that might influence their decisions and conclusions around a particular piece of content. In the interests of transparency and to help readers form their own judgements of potential bias, Nature Portfolio journals require authors to declare any competing financial and/or non-financial interests in relation to the work described.

Please provide a 'Competing interests' statement using one of the following standard sentences:

1. The authors declare the following competing interests: [specify competing interests]
2. The authors declare no competing interests.

See the Nature Portfolio competing interests policy for further information:
https://www.nature.com/nature-research/editorial-policies/competing-interests
The Springer Nature policy can be found here:

We believe that Springer Nature has a responsibility to support the relevant guidelines (based on research community or geographical region) that specify best practice in research and thus require all experimental results on animal and human participants to conform to the authors' local regulations and ethical standards, and we also encourage adherence to international standards.

Because your study uses live vertebrates, a statement affirming that you have complied with all relevant ethical regulations for animal testing and research is necessary. A statement explicitly confirming if the study received ethical approval, including the name of the board and institution that approved the study protocol is also required. The species, strain, sex and age of animals should be included.

Further details on our policies can be found at
https://www.nature.com/commsbio/editorial-policies/ethics-and-biosecurity

Reporting & reproducibility

Please state in the legends how many times each experiment was repeated independently with similar results. This is needed for all experiments, but is particularly important wherever results from representative experiments (such as micrographs) are shown. If space in the legends is limiting, this information can be included in a section titled "Statistics and Reproducibility" in the methods section.

Statistical reporting
Wherever statistics have been derived (e.g. error bars, box plots, statistical significance) the legend needs to provide and define the n number (i.e. the sample size used to derive statistics) as a precise value (not a range), using the wording "n=X biologically independent samples/animals/cells/independent experiments/n= X cells examined over Y independent experiments" etc. as applicable.
Statistics such as error bars, significance and p values cannot be derived from n<3 and must be removed from all such cases.
We strongly discourage deriving statistics from technical replicates, unless there is a clear scientific justification for why providing this information is important. Conflating technical and biological variability, e.g., by pooling technical replicates samples across independent experiments is strongly discouraged. (For examples of expected description of statistics in figure legends, please see the following https://www.nature.com/articles/s41467-019-11636-5 or https://www.nature.com/articles/s41467-019-11510-4).
All error bars need to be defined in the legends (e.g. SD, SEM) together with a measure of centre (e.g. mean, median). For example, the legends should state something along the lines of "Data are presented as mean values +/- SEM" as appropriate.<br><br>All box plots need to be defined in the legends in terms of minima, maxima, centre, bounds of box and whiskers and percentile.<br><br>**Legends requiring revision:**<br>1. If the shaded areas denote error bands then the error bands need to be defined in the legends of figures 2b; 4a-e.
The figure legends must indicate the statistical test used. Where appropriate, please indicate in the figure legends whether the statistical tests were one-sided or two-sided and whether adjustments were made for multiple comparisons.<br><br>For null hypothesis testing, please indicate the test statistic (e.g. F, t, r) with confidence intervals, effect sizes, degrees of freedom and P values noted.<br><br>Please provide the test results (e.g. P values) as exact values whenever possible and with confidence intervals noted.<br><br>**Legends requiring revision:**<br>1. Please indicate the statistical test used for data analysis and where appropriate, please specify whether it was one-sided or two-sided and whether adjustments were made for multiple comparisons, in the legends of supplementary figures 1c, d.

The quality of some of the figures appears to be quite low. If possible, we suggest replacing these with higher-resolution images.
**Other notes**
Please note that the legend for Figure 2c is incorrectly labelled as '2d'. Please rectify this in the figure legend.<br><br>Please also note that Supplementary Table 8 is incorrectly labelled as 'Supplementary Table 1'. Please rectify this in the Supplementary Tables file.
