# Supplementary material for: Chromosome-scale genome assembly of the brown anole (Anolis sagrei), an emerging model species
Source: Commun Biol. 2022 Oct 25;5:1126. doi: 10.1038/s42003-022-04074-5 (PMC9596491; doi:10.1038/s42003-022-04074-5)
Supplement: Supplementary file 3 — Description of Additional Supplementary Files [file 42003_2022_4074_MOESM3_ESM.docx]

Description of Additional Supplementary Files

**Supplementary Data 1.** Publications per year mentioning *Anolis sagrei* or *A. carolinensis****.***

**Supplementary Data 2.** Completeness and Contiguity of preliminary and final assemblies of *Anolis sagrei*

**Supplementary Data 3.** Completeness and contiguity of *Anolis sagrei* and other lepidosaur genome assemblies

**Supplementary Data 4.** Annotation Summary Statistics

**Supplementary Data 5**. Anolis sagrei Jukes/Cantor and Kimura substitution levels adjusted for CpG sites. Weighted average Kimura divergence for each repeat family

**Supplementary Data 6.** A carolinensis Jukes/Cantor and Kimura substitution levels adjusted for CpG sites. Weighted average Kimura divergence for each repeat family

**Supplementary Data 7.** Chromosomes assigned to chromosome 13 (the ancient X) in *A. carolinensis* in previous publications and inferred in this study. Checked boxes represent scaffolds found in given study.

**Supplementary Data 8.** Sequency homology between AnoSag2.1 scaffold 7 and AnoCar2.0 as inferred by Satsuma

**Supplementary Data 9.** Sampling information for *A. sagrei* used for sex chromosome identification.

**Supplementary Data 10.** Position of *A. sagrei* homologs of *A. carolinensis* X-linked genes

**Supplemental Data 11.** Summary of X chromosome synonymous and non-synonymous sites as well as genes per 50kb sliding windows.

**Supplemental Data 12.** Summary of X chromosome SNPs and Indels per 10kb sliding window.

**Supplemental Data 13.** X chromosome GWAS results for male/female identity

**Supplementary Data 14.** Raw sequencing read data used for assembly

**Supplementary Data 15.** Comparison of AnoSag2.1 scaffold size and chromosome sizes estimated using measurements of chromosomes imaged in Figure 1C, Giovannotti et al. 2017

**Supplementary Data 16.** Window analysis of ddRAD inferred SNPs
